# Effect of neuron‐derived neurotrophic factor on rejuvenation of human adipose‐derived stem cells for cardiac repair after myocardial infarction

**DOI:** 10.1111/jcmm.14456

**Published:** 2019-07-09

**Authors:** Kun Yang, Hui‐Fang Song, Sheng He, Wen‐Juan Yin, Xue‐Mei Fan, Feng Ru, Hui Gong, Xiao‐Yan Zhai, Jie Zhang, Ze‐Xu Peng, Guang‐Xia Xi, Jun Xie, Ren‐Ke Li

**Affiliations:** ^1^ Department of Biochemistry and Molecular Biology, Shanxi Key Laboratory of Birth Defect and Cell Regeneration Shanxi Medical University Taiyuan China; ^2^ Department of Endocrinology Shanxi Dayi Hospital affiliated to Shanxi Medical University Taiyuan China; ^3^ Department of Anatomy Shanxi Medical University Taiyuan China; ^4^ Department of Radiology The First Hospital of Shanxi Medical University Taiyuan China; ^5^ Department of Urology The First Hospital of Shanxi Medical University Taiyuan China; ^6^ Department of Anatomy Shanxi University of Chinese Medicine Yuci China; ^7^ Division of Cardiovascular Surgery, Toronto General Research Institute University Health Network Toronto Ontario Canada; ^8^ Division of Cardiac Surgery, Department of Surgery University of Toronto Toronto Ontario Canada

**Keywords:** ageing, cardiac injury repair, human adipose‐derived stem cells, NDNF, rejuvenation

## Abstract

The decline of cell function caused by ageing directly impacts the therapeutic effects of autologous stem cell transplantation for heart repair. The aim of this study was to investigate whether overexpression of neuron‐derived neurotrophic factor (NDNF) can rejuvenate the adipose‐derived stem cells in the elderly and such rejuvenated stem cells can be used for cardiac repair. Human adipose‐derived stem cells (hADSCs) were obtained from donors age ranged from 17 to 92 years old. The effects of age on the biological characteristics of hADSCs and the expression of ageing‐related genes were investigated. The effects of transplantation of NDNF over‐expression stem cells on heart repair after myocardial infarction (MI) in adult mice were investigated. The proliferation, migration, adipogenic and osteogenic differentiation of hADSCs inversely correlated with age. The mRNA and protein levels of NDNF were significantly decreased in old (>60 years old) compared to young hADSCs (<40 years old). Overexpression of NDNF in old hADSCs significantly improved their proliferation and migration capacity in vitro. Transplantation of NDNF‐overexpressing old hADSCs preserved cardiac function through promoting angiogenesis on MI mice. NDNF rejuvenated the cellular function of aged hADSCs. Implantation of NDNF‐rejuvenated hADSCs improved angiogenesis and cardiac function in infarcted mouse hearts.

## 1 INTRODUCTION

Global coronary heart disease, especially acute myocardial infarction (MI), is associated with high morbidity and mortality, and the age of first MI is decreasing.^1^ The cost of healthcare continues to rise as the size of the aged population increases, contributing to major social and economic problems worldwide.^2^, ^3^ Advancements in clinical treatments including thrombolytic drugs, coronary artery bypass grafts and interventional treatments have greatly improved the prognosis of MI. However, progression to congestive heart failure remains a significant global health crisis, especially in aged populations. Since current therapies focus on the restoration of blood flow to the defect area, the damaged cardiomyocytes are unable to regenerate and are replaced by fibroblasts and collagen matrix (fibrotic scar tissue). This scar tissue becomes thin and inelastic, leading to ventricular chamber dilation and progression to congestive heart failure.^4^

In recent years, with the rapid development of tissue engineering and regenerative medicine, stem cell therapy for MI has provided new hope and ideas for cardiac repair. Autologous stem cell transplantation has greatly reduced the problem of immune rejection and has become one of the most promising treatment methods at present.^5^, ^6^, ^7^ Currently, stem cells used for tissue repair and regeneration include embryonic stem cells, hematopoietic stem cells, bone marrow mesenchymal stem cells and adipose‐derived stem cells (ADSCs). All these cells have entered the clinical trial stage in the fields of soft tissue defects, ischaemic heart disease, diabetes and neural degenerative diseases.^8^, ^9^ ADSCs have become more popular cells in tissue repair because of their high abundance and relative ease of extraction. They also induce a low immune response in the host and have multi‐directional differentiation and self‐renewal abilities.^10^, ^11^, ^12^ In the current study, we focus on ADSC transplantation for the treatment of acute MI and investigated its efficacy for cardiac repair and regeneration.

Clinical studies found that the efficacy of stem cell therapy in elderly patients is significantly reduced.^13^ Although there are multiple contributing factors, age is the most important. With ageing, the number and quality of stem cells significantly decrease, and their ability to repair and regenerate is compromised. This decline in stem cell function results in the unsatisfactory clinical effect of autologous stem cell transplantation in aged patients.^14^

Neuron‐derived neurotrophic factor (NDNF, also named C4orf31) is identified as a secretory protein which promotes the growth and migration of neurons, the formation of extracellular matrix and inhibits apoptosis.^15^, ^16^ Previously, our group found that NDNF protein rejuvenated aged human multipotent mesenchymal stromal cells (hMSCs) and transplantation of the NDNF‐rejuvenated hMSCs improved cardiac function through inhibiting apoptosis and promoting angiogenesis in infarcted mouse hearts.^17^ However, the effect of age on the expression level of NDNF in human adipose‐derived stem cells (hADSCs) remains unknown and studies on the treatment of MI with NDNF overexpression by hADSCs have not been reported. We have been suggested that NDNF can be selected as a target to rejuvenate aged hADSCs through genetic modification, thereby improving the ability of aged stem cells to facilitate angiogenesis and tissue repair and ultimately lead to the preservation in cardiac function. We used a lentiviral vector to overexpress NDNF in old hADSCs and the biological function of proliferation and migration of hADSCs was investigated in vitro. The genetically modified aged stem cells were then implanted into heart after MI using an adult mouse model and the effects of NDNF overexpression hADSCs on heart repair were investigated in vivo.

## 2 MATERIALS AND METHODS

### 2.1 Human adipose tissue collection, cultivation and identification

Adipose tissue was obtained from hospitalized patients (17‐92 years old) who underwent abdominal surgery (including caesarean section, hepatectomy, cholecystectomy, splenectomy, etc) in general surgery, obstetrics and gynaecology and urology in the First Hospital of Shanxi Medical University. All specimens acquired were obtained with the patient's informed consent and the study was approved by the hospital's ethics committee. Patients who were positive for infectious diseases, systemic diseases or malignancy, were excluded. The patients were randomly selected in different experiments for different genders, to ensure that there is no statistical difference for that characteristic. The general characteristics of the patients for in vitro and in vivo studies are listed in Table S1. For all the in vitro studies, hADSCs from single donors were used for each evaluation. The N number (equal to patient numbers) for each experiment was indicated in the figure legends.

Adipose tissue was obtained under aseptic conditions; the blood was washed, blood vessels and fascia removed, cut into pieces, and digested with an equal volume of 0.1% type I collagenase. It was transferred to a water bath thermostat oscillator for digestion for 60 min (37°C, 100 rpm). It was then neutralized with Dulbecco's Modified Eagle's Medium/Ham's Nutrient Mixture F‐12 (DMEM/F12, Gibco, cat no. 12400024) containing 10% FBS in equal volume. The tissue homogenate was filtered and centrifuged at 450 *g* for 8 minutes. The cell suspension was counted with a cell counting plate and inoculated with 1‐2 × 10^4^ cells in 25 mm^2^ culture dish. This was followed by incubation at 37°C with 5% CO_2_ in a cell incubator. The cell culture medium was changed after 24 hours, and then again 2‐3 days later. When the cells reached 80%‐90% confluence, they were passaged for expansion. Morphological observation was made using an inverted microscope.

For cell identification with flow cytometry, hADSCs cultured until passage 3 were digested with trypsin and centrifuged. The cells were divided into young (<40 years) and old (>60 years) groups. One million cells from each sample were taken for antibody staining with cell surface markers or isotype‐identical IgG (FITC Mouse Anti‐Human CD90, cat no. 51‐9007657; PE Mouse Anti‐Human CD44, cat no. 51‐9007656; APC Mouse Anti‐Human CD73, cat no. 51‐9007649; PerCP‐Cy^TM^5.5 Mouse Anti‐Human CD105, cat no. 51‐9007648; PE hMSC Negative Cocktail, cat no. 51‐9007661; all from BD Biosciences) for half an hour. The cells were then washed and resuspended in PBS supplemented with 2% foetal bovine serum (FBS) and 0.1% sodium azide. Cells were analysed using a Becton Dickinson LSRII flow cytometer. The fluorescence intensity of 10 000 cells for each sample was quantified.

### 2.2 Overexpression of NDNF in old hADSCs by gene modification

Cell transduction was carried out using a lentiviral expression vector carrying the NDNF gene (Lenti‐Puro‐EF1α‐ NDNF‐Homo‐ IRES‐eGFP, Cyagen Biosciences Inc, Santa Clara, CA) according to the manufacturer's instructions. Empty virus (Old) and NDNF (Old + NDNF) were transduced into old hADSCs by lentiviral vector (n = 6, age 72.5 ± 10.52 years). The expression differences of mRNA and protein levels of NDNF after transduction were detected by RT‐PCR and Western blotting as described in supplemental methods. The effect of overexpression of NDNF on cell proliferation and migration was observed by BrdU (5‐bromo‐2'‐deoxyuridine, Sigma, cat no. A2385) pulse chasing and the wound‐healing cell migration assay described in supplemental methods.

### 2.3 Myocardial infarction model

Female C57BL/6 mice from the Laboratory Animal Center of Shanxi Medical University (20‐25 g at 2–month‐old) were used for the procedures. All animal experiments were conducted in accordance with the Guide for the Care and Use of Laboratory Animals (NIH, revised 2011). Mice were divided into four groups according to the different types of injected cells, including control group receiving medium injection (Medium), old hADSCs transduced by empty virus (Old), old hADSCs that overexpressed NDNF (Old + NDNF) and young hADSCs (Young). For the in vivo transplantation study, hADSCs were obtained from seven young (Young, age 30.86 ± 4.45 years old) and seven old (Old, age 72.14 ± 9.65 years old) individual patients. Cells derived from individual patient from the young or the old group were respectively used to inject 3‐4 mice from each experimental group. Mice were anaesthetized and intubated using 2% isoflurane. Permanent ligation of the left anterior descending coronary artery was performed to induce MI, and the infarcted area was controlled between 30% and 35% of the left ventilated free wall. One million cells in 20 μL serum‐free DMEM/F12 medium were injected into the border zone of the infarcted area for each mouse. Cyclosporine A (5 mg/kg) was injected intraperitoneally every day until the end‐point of the experiments at 28 days after MI.

### 2.4 Cardiac function measurement

Echocardiograph was used to dynamically record the changes in cardiac function of mice. The left ventricular internal dimension in systole (LVIDs), left ventricular internal dimension‐diastole (LVIDd), ejection fraction (EF%) and left ventricular fractional shortening (FS%) of mice were measured before and 7, 14, 21 and 28 days after MI. Twenty‐eight days after MI and cell transplantation, the mouse hearts were dissociated from surrounding tissue. After fixation with 10% formalin for 48 hours and dehydration with 75% ethanol, the hearts were cut along the horizontal axis into continuous 1 mm sections, photographed for morphometry. ImageJ software was used to measure the size of the infarcted area (the ratio between the length of the infarct area and the circumference of the entire left ventricle) and the thickness of the infarct respectively.

### 2.5 Masson's trichrome staining

The heart segments were embedded in paraffin and made into 5‐μm thick sections. The level of cardiac fibrosis was detected by Masson's trichrome staining. Briefly, after gradient dewaxing and fixation, the paraffin sections were successively immerged into different colours of dyes. The red coloured area represented viable cardiac tissue and the blue coloured area represented collagen fibres, which was performed to confirm scar tissue in the left ventricular free wall.

### 2.6 Immunofluorescent staining

Von Willebrand factor (vWF) and α‐smooth muscle actin (α‐SMA) were detected by immunofluorescence staining to compare differences in the number of new capillaries and small arterioles. After the gradient dewaxing of paraffin sections, slides were incubated in Tris‐EDTA by microwave heating for 10 minutes, and subsequently blocked in BSA for 1 hour. Primary antibodies (rabbit anti‐vWF, Proteintech, cat no. 11778‐1‐AP at 1:100 dilution, mouse anti‐α‐SMA, Sigma, cat no. A5228 at 1:200 dilution) were incubated at 4°C overnight. The next day, incubation with Alexa568 secondary antibody (goat anti‐rabbit, Invitrogen, cat no. A11011) and Alexa488 secondary antibody (rabbit antimouse, Invitrogen, cat no. A11029) at 1:2000 dilution was carried out for 1 hour at room temperature. The nuclei were counter stained with DAPI (Sigma, cat no. D9542) for 10 min. The fluorescent‐positive area in three randomly selected high‐power fields per slide was photographed using a Nikon fluorescence inverted microscope. ImageJ software was used to calculate the percentage of fluorescent positive areas.

### 2.7 Data analysis

All data are presented as mean ± SD. Statistical analyses were performed with GraphPad Prism software (v.7.0). Student's *t* test was used for comparisons of means between two groups. Comparisons of parameters among three or more groups were analysed using one‐way ANOVA followed by Tukey or two‐way ANOVA with repeated measures over time, followed by Bonferroni *post‐hoc* tests. The list of X (age) and Y (proliferation/migration/ differentiation level/mRNA levels of the 7 factors) was analysed using a correlation analysis. Differences were considered statistically significant at *P* < 0.05.

## 3 RESULTS

### 3.1 HADSCs were successfully isolated, cultured and identified

**Figure 1:**
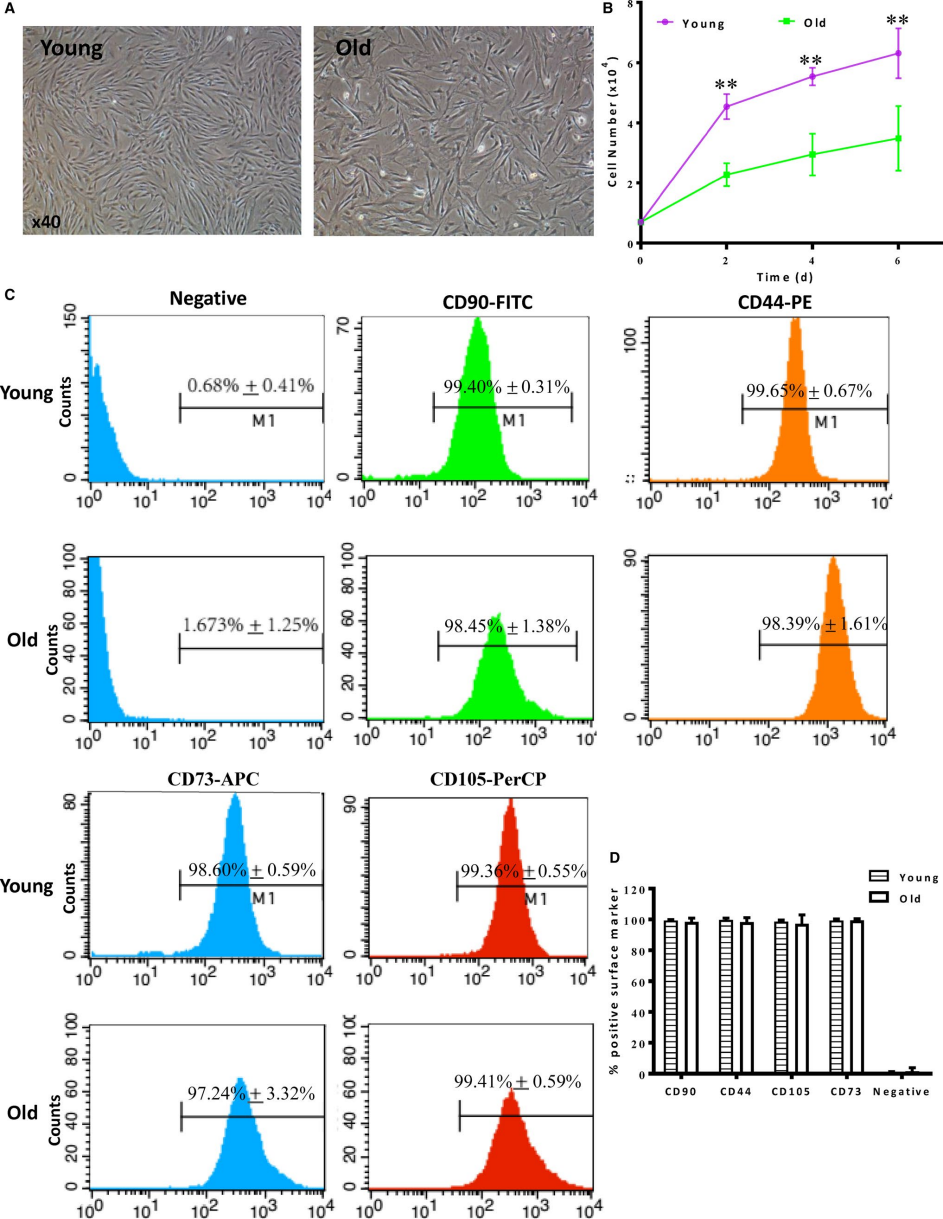
Cultivation and identification of hADSCs. A, General morphological observation of human adipose‐derived stem cells (hADSCs) cultured for 7 days after isolation from donors (17‐92 years old). B, Rate of cell growth was significantly higher in young than old hADSCs, n = 6/group, **P < 0.01 Young vs Old. C, Representative histogram plots of cell surface markers from young and old hADSCs. D, Comparison of % positive cells of hADSCs from young and old donors. n = 3/group

Human adipose‐derived stem cells were successfully isolated and the cell morphology was identified under a microscope. HADSCs in the young group were long fusiform, growing radially or vertically around each centre, with uniform shape, size and an orderly arrangement. However, hADSCs in the old group were spindle or polygonal in shape and arranged in a non‐uniform way (Figure 1A). Next, we seeded the cells at the same number and counted at day 2, 4 and 6 after cell plating to determine the growth curve. The growth of young hADSCs was significantly higher than those of the old hADSCs starting from day 2 and sustained up to day 6 after cell seeding (Figure 1B). The hADSCs from both young and old groups were more than 95% positive for the ADSC surface markers (CD90, CD44, CD73 and CD105) as identified by flow cytometry (Figure 1C and 1D). On the other hand, the hADSCs from both groups were negative for the haematopoietic lineage cell markers of CD45, CD11b, CD19 and HLA‐DR (Data not shown). These results conform to the criteria of ADSC identification.

### 3.2 Proliferation, migration, adipogenic and osteogenic differentiation of hADSCs decreased with age

**Figure 2:**
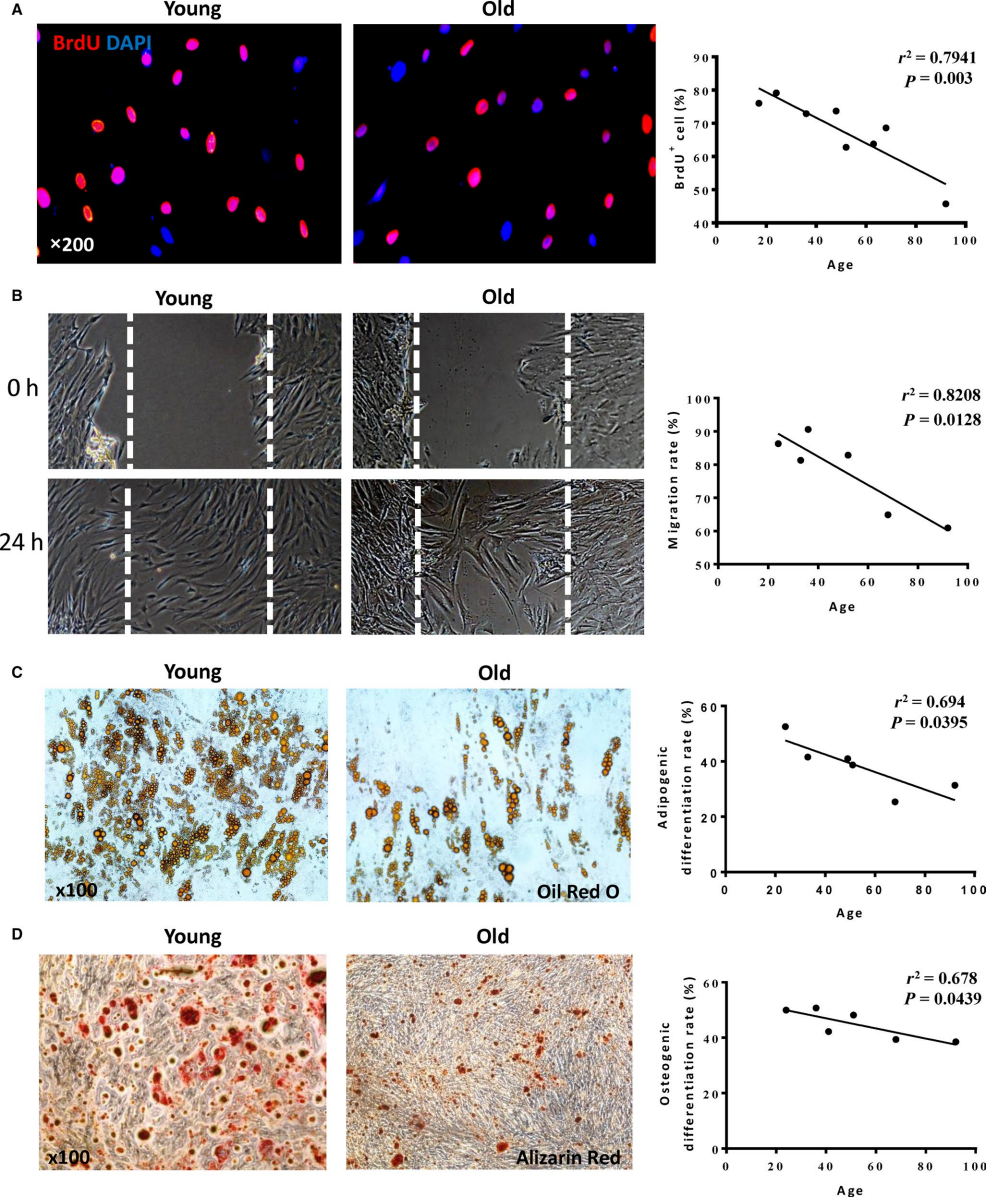
The proliferation, migration and differentiation of hADSCs decreased with age. A, Representative micrographs of immunofluorescent staining for 5‐bromo‐2'‐deoxyuridine (BrdU, red) with nuclei stained blue with DAPI. The percentage of BrdU^+^ cells decreased with age. B, Representative images showed the cell migration of human adipose‐derived stem cells (hADSCs) using the wound‐healing cell migration assay. Migration rate decreased with age. C, Representative micrographs of adipogenic differentiation stained for oil red ‘O’. Mature adipose cells were stained in red in both old and young groups. The adipogenic differentiation rate decreased with age. D, Representative micrographs of osteogenic differentiation stained for alizarin red. The osteocytes were stained in red in both old and young groups. The osteogenic differentiation rate decreased with age

To further confirm the difference in cell growth capacity between the young and old hADSCs, we performed BrdU pulse chasing to label actively proliferating cells. Consistent with the result from cell growth, there was an inverse correlation between the number of BrdU^+^ cell and age showing decreasing number of BrdU^+^ cells with increasing age (Figure 2A). On testing the cell migratory ability by wound‐healing cell migration assay, the result revealed that the migration ability of hADSCs decreased with age (Figure 2B).

Adipogenic and osteogenic differentiation can be induced in hADSCs, and a large number of cells were stained by oil red O and alizarin red, which confirmed that this cell had adipogenic and osteogenic differentiation abilities. However, the adipogenic (Figure 2C) and osteogenic (Figure 2D) differentiation abilities gradually declined with age.

### 3.3 The mRNA and protein levels of NDNF decreased significantly with age

**Figure 3:**
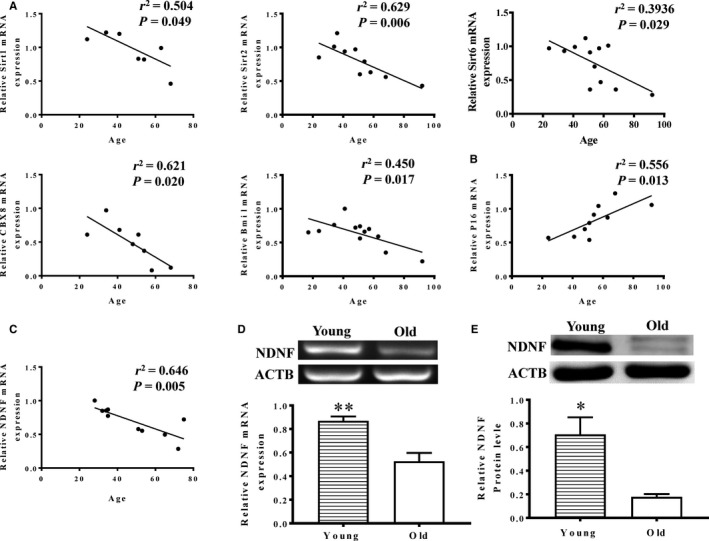
NDNF expression decreased with age. A, The cell regeneration‐related genes, Sirt1 (sirtuin), Sirt2, Sirt6, Bmi1 (Polycomb complex protein BMI‐1), and Cbx8 (chromobox homolog 8), in human adipose‐derived stem cells (hADSCs) negatively correlated with age. B, The cell senescence‐related gene P16 (cyclin‐dependent kinase inhibitor 2A) increased with age. C, Neuron‐derived neurotrophic factor **(**NDNF) mRNA expression showed inverse correlation with age. The mRNA (D, n = 5/group) and protein (E, n = 3/group) levels of NDNF in the elderly group was significantly lower than that of the youth group. *P < 0.05, **P < 0.01

In an attempt to identify the possible factor (factors) responsible for the age‐related changes in cellular function, we performed an array of RT‐PCR to examine the regeneration‐ and senescence‐related genes. There was a significant negative correlation with age among regeneration‐related genes Sirt1 (sirtuin), Sirt2, Sirt6, Cbx8 (chromobox homolog 8) and Bmi1 (Polycomb complex protein BMI‐1, Figure 3A) whereas the cell senescence‐related gene P16 (cyclin‐dependent kinase inhibitor 2A) increased with age (Figure 3B). Among these genes, the NDNF mRNA showed a strong inverse correlation with age (Figure 3C). This was further confirmed by the result showing that the mRNA (Figure 3D) and protein (Figure 3E) levels of NDNF in the elderly group were significantly lower than that of the youth group. These findings implied that NDNF may be one of the key factors involved in age‐related changes in hADSC function.

### 3.4 Overexpression of NDNF in old hADSCs significantly enhanced cell proliferation and migration

**Figure 4:**
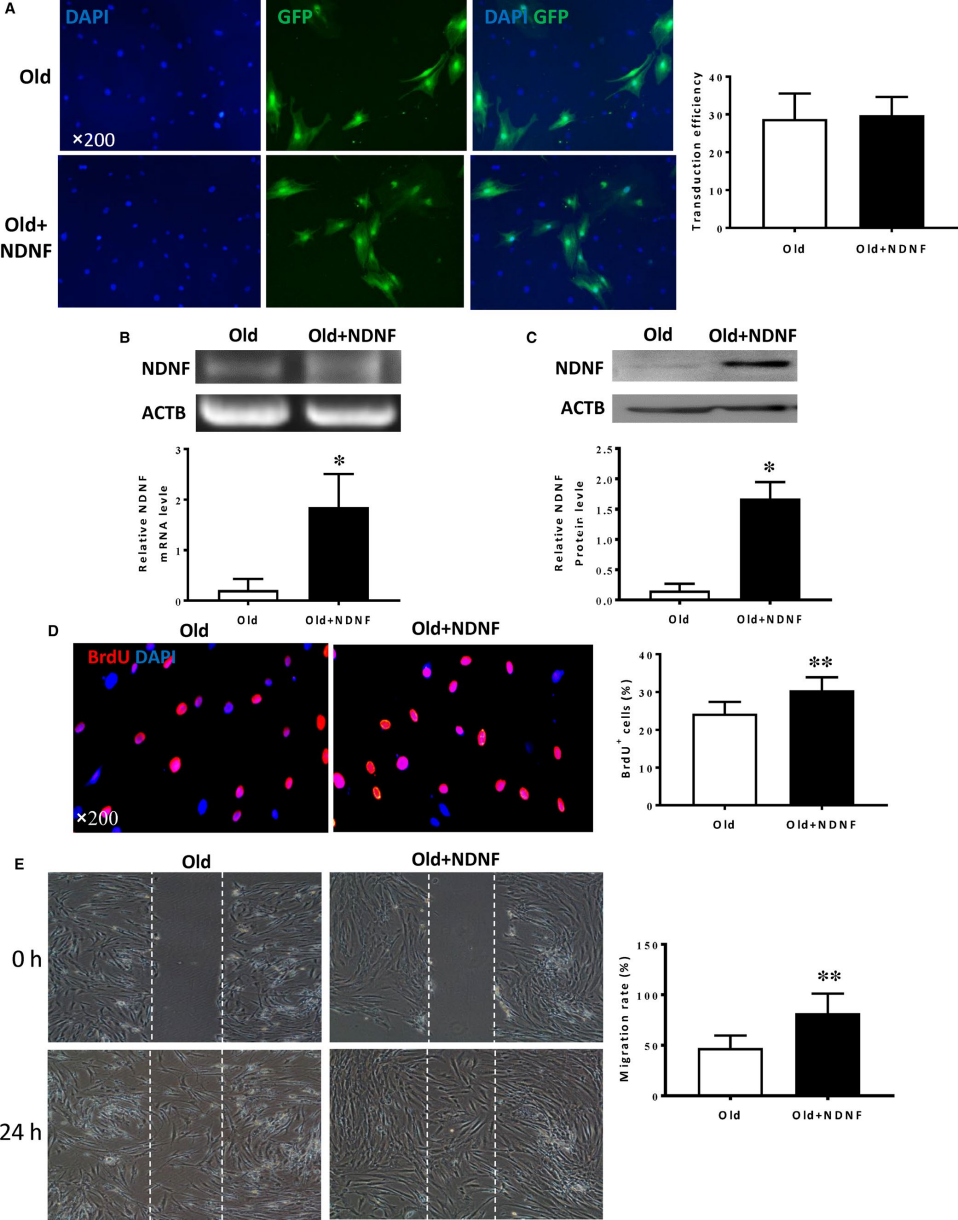
NDNF transduction rejuvenated old hADSCs by increasing proliferation and migration. A, Old human adipose‐derived stem cells (hADSCs) were transduced with a lentiviral vector overexpressing neuron‐derived neurotrophic factor (NDNF) which was also tagged with green fluorescent protein (GFP). Representative micrographs showed the GFP^+^ cells to indicate the transduction efficiency. There was no difference in transduction efficiency between old hADSCs transduced with NDNF (Old + NDNF) or with empty viruses (Old), n = 6/group. B, NDNF mRNA expression evaluated by RT‐PCR was significantly higher in Old + NDNF compared to Old group, n = 3/group. C, NDNF protein expression evaluated by Western blotting was significantly higher in Old + NDNF compared with that of the Old group, n = 3/group. D, Representative micrographs of immunofluorescent staining for 5‐bromo‐2'‐deoxyuridine (BrdU in red) with nuclei stained blue with DAPI. The percentage of BrdU^+^ cells was significantly higher in Old + NDNF compared with that of the Old group, n = 6/group. E, Representative images showed cell migration of hADSCs using the wound‐healing cell migration assay. Migration rate was significantly higher in Old + NDNF compared with empty vector‐transduced hADSCs (Old), n = 6/group. *P < 0.05, **P < 0.01

To investigate if restoring NDNF level can restore the proliferative and migratory capabilities in old hADSCs, a lentiviral expression vector carrying the NDNF gene (also tagged with green fluorescent protein, GFP) was used to transduce old hADSCs (Old + NDNF). Old hADSCs transduced with empty vector without the NDNF gene but with GFP served as control (Old). Transduction efficiency when quantified by GFP^+^ cells was comparable between the two groups (Figure 4A). The expression of NDNF mRNA (Figure 4B) and protein (Figure 4C) was significantly greater in NDNF‐transduced than in empty vector‐transduced old hADSCs. As expected, BrdU pulse chasing revealed that the number of BrdU^+ ^cells in Old + NDNF were significantly higher than that of the old group (Figure 4D). The cell migration test showed that the migration rate in Old + NDNF was significantly greater compared with that of the old group (Figure 4E). These data confirmed our hypothesis that restoration of NDNF improved proliferation and migration of old hADSCs in vitro.

### 3.5 NDNF promoted the repair of cardiac injury in vivo

**Figure 5:**
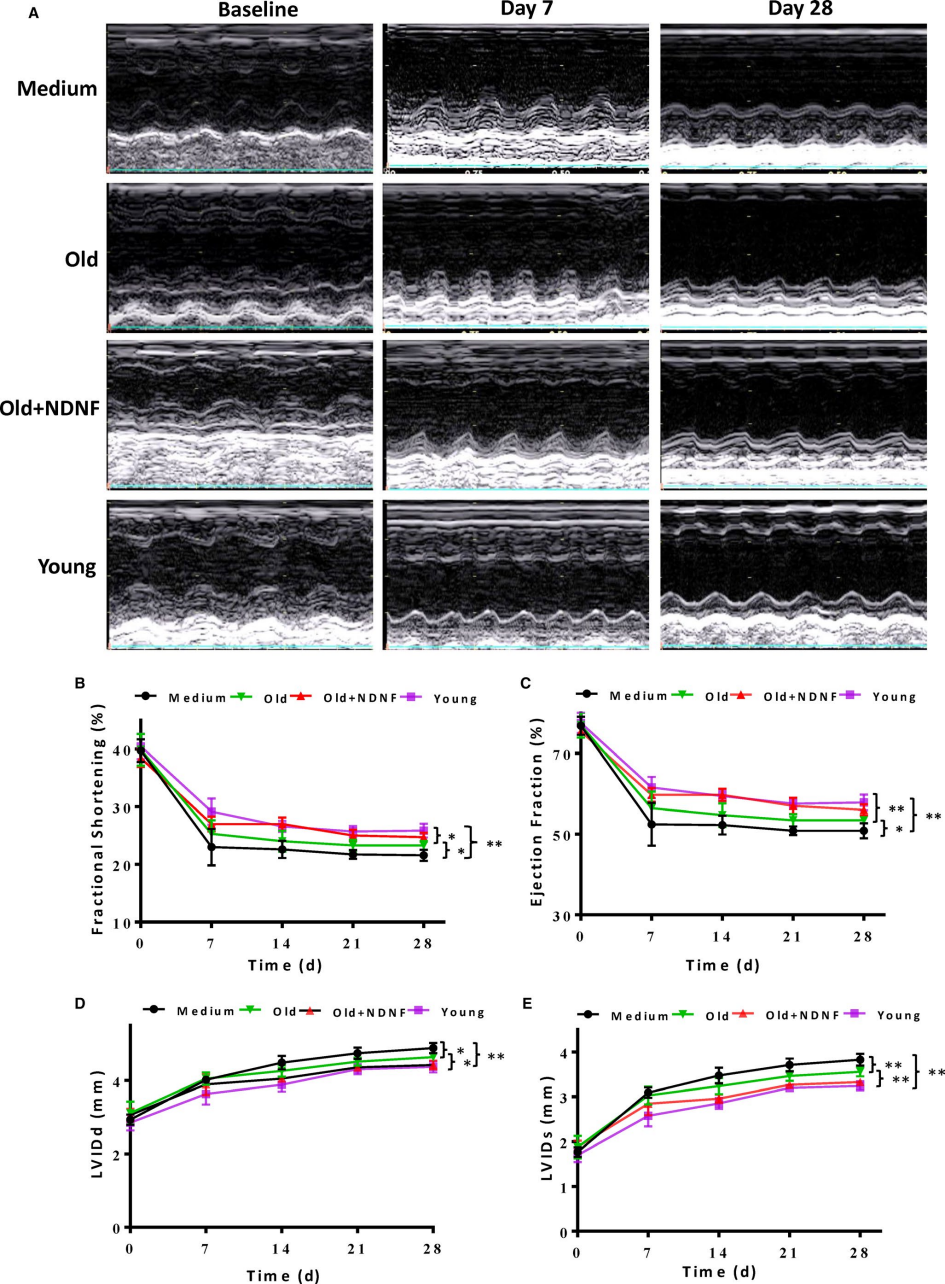
In vivo implantation of NDNF‐overexpressing old hADSCs improved cardiac function after MI. A, Representative M‐mode echocardiographic images taken before (baseline), and 7 and 28 days after myocardial infarction (MI) in mice that received implantation of control medium (Medium), empty vector‐transduced old human adipose‐derived stem cells (hADSCs, Old), neuron‐derived neurotrophic factor (NDNF)‐transduced old hADSCs (Old + NDNF) and untransduced young hADSCs (Young). The left ventricular fractional shortening (B, FS %), ejection fraction (C, EF %), left ventricular internal dimension‐diastole (D, LVIDd) and left ventricular internal dimension in systole (E, LVIDs ), of mice before and 7, 14, 21, and 28 days after MI, reflecting the changes in cardiac function of mice. FS and EF were significantly higher whereas LVIDs and LVIDd were significantly lower in the Young and Old + NDNF compared to the Old and Medium groups at 28 days after MI. n = 7/group, *P < 0.05,**P < 0.01

To further examine the reparative capacity of NDNF‐overexpressed old hADSCs, a mouse model with left coronary artery ligation to induce MI was used. Cardiac function was determined by echocardiography in mice that received implantation of control medium (Medium), empty vector‐transduced old hADSCs (Old), NDNF‐transduced old hADSCs (Old + NDNF) or untransduced young hADSCs (Young) into the border region immediately following MI. M‐mode echocardiographic images were taken before and 7 and 28 days after MI (Figure 5A). The results showed that baseline function before MI was similar among the four groups with respect to FS (Figure 5B), EF (Figure 5C), Left Ventricular Internal Dimension‐diastole (LVIDd, Figure 5D) and Left Ventricular Internal Dimension in systole (LVIDs, Figure 5E). After MI, FS and EF were significantly higher in the Old + NDNF than the medium and the old groups with the highest level in the young group. On the other hand, LVIDs and LVIDd were significantly lower in the Old + NDNF group than in the medium and the old groups with the lowest level in the young group post‐MI. The same trend sustained for up to 4 weeks for all the four groups. These results indicated that implantation of NDNF‐overexpressed old hADSCs partially restored cardiac function in infarcted mouse hearts.

**Figure 6:**
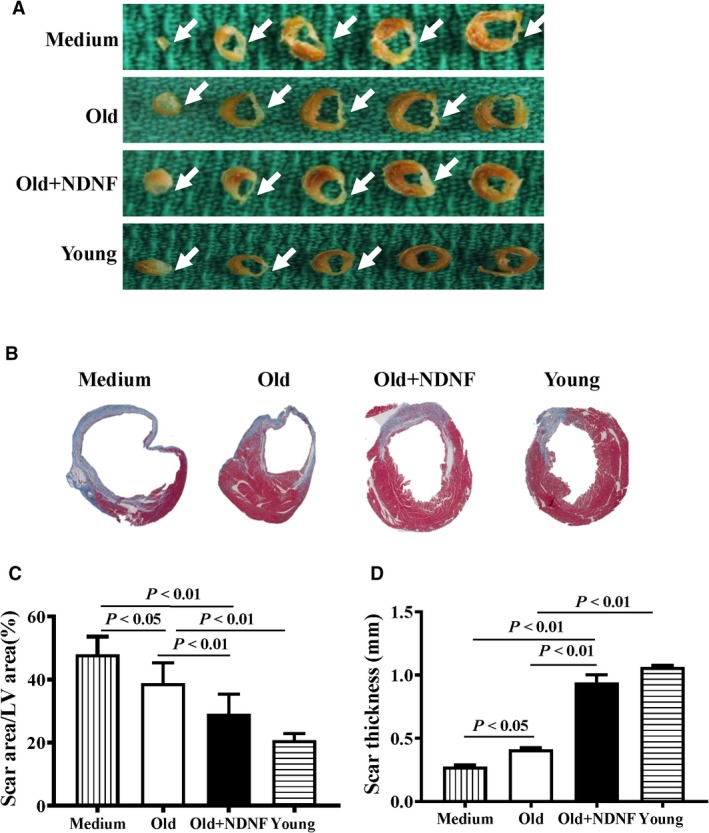
In vivo implantation of NDNF‐overexpressing old hADSCs decreased scar size and increased scar thickness in infarcted mouse hearts. A, Representative heart sections 28 days after myocardial infarction (MI) in mice that received implantation of control medium (Medium), empty vector‐transduced old human adipose‐derived stem cells (hADSCs, Old), neuron‐derived neurotrophic factor (NDNF)‐transduced old hADSCs (Old + NDNF) and untransduced young hADSCs (Young). B, Representative heart slides stained with Masson's trichrome and planimetry‐based quantification revealed that the scar size area was larger in the Medium and Old groups than in the Old + NDNF and Young groups at 28 days after MI (C). D, The scar thickness was greater in the Old + NDNF and Young groups than in the Medium and Old groups at 28 days after MI, n = 6/group

**Figure 7:**
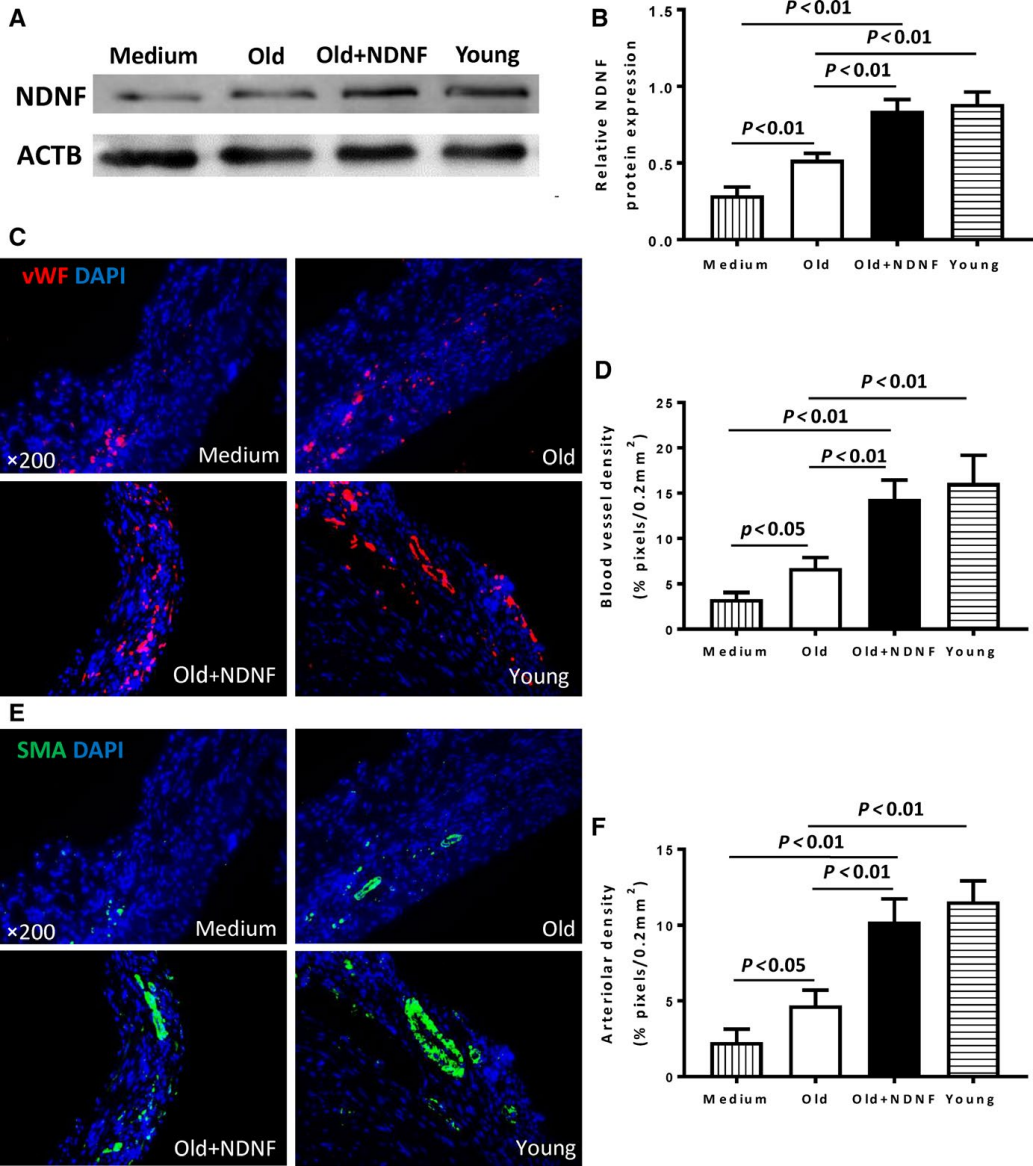
In vivo implantation of NDNF‐overexpressing old hADSCs increased angiogenesis and arteriole genesis. A, Western blotting analysis of the expression of NDNF protein in the infarcted area of mouse hearts that received implantation of control medium (Medium), empty vector‐transduced old human adipose‐derived stem cells (hADSCs, Old), neuron‐derived neurotrophic factor (NDNF)‐transduced old hADSCs (Old + NDNF) and untransduced young hADSCs (Young). B, NDNF protein levels were significantly higher in the Old + NDNF and Young groups compared with the Old and Medium control groups, n = 3/group. C, Representative micrographs of immunofluorescent staining for von Willebrand factor (vWF, red) with nuclei stained blue with DAPI. D, Capillary density was significantly higher in the Old + NDNF and Young groups compared with the Old and Medium control groups, n = 6/group. E, Representative micrographs of immunofluorescent staining for α‐smooth muscle actin (α‐SMA, green) with nuclei stained blue with DAPI. F, Arteriole density was significantly higher in the Old + NDNF and Young groups compared with the Old and Medium control groups, n = 6/group

Morphological analysis (Figure 6A) and Masson's trichrome staining of hearts (Figure 6B) indicated that the scar size in the Old + NDNF was significantly smaller than that in the medium and the old groups with the young group had the smallest scar size at 28 days post‐MI (Figure 6C). On the other hand, scar thickness was significantly greater in the Old + NDNF than that in the medium and the old groups with the young group had the greatest scar thickness (Figure 6D). Next, to confirm that implantation of NDNF‐overexpressing hADSCs‐restored NDNF level in the infarcted hearts in vivo, the protein level of NDNF was examined in the infarct and border areas from the four experimental groups (Figure 7A). The protein expression of NDNF as measured by Western blotting was significantly higher in the infarcted area of the Old + NDNF than in the medium control and the old groups (Figure 7B). The protein expression of NDNF in the Old + NDNF was comparable to that of the young group, indicating implantation of NDNF‐overexpressing hADSCs effectively restored NDNF levels in the infarcted hearts (Figure 7B). To further understand the underlying mechanism responsible for the functional changes, immunofluorescent staining was performed to determine blood vessel density (stained with vWF) and arteriole density (stained with α‐SMA). The results showed that blood vessel density (Figure 7C and D) and arteriole density (Figure 7E and 7F) of the young and the Old + NDNF groups were significantly greater than those of the medium control group and the old group with empty virus transduction. Collectively, these data suggested that implantation of NDNF‐overexpressing old hADSCs promoted cardiac repair and delayed progressive heart failure possibly through restoration of NDNF level and improving angiogenesis.

## 4 DISCUSSION

In the current study, hADSCs were successfully isolated from young and old donors with the expression of surface markers conform to the criteria of stem cell identification. NDNF was identified as one of the key factors down‐regulated in the aged hADSCs. Restoration of NDNF in the aged hADSCs was achieved with gene overexpression. Restoration of NDNF improved the proliferative and migratory abilities of the aged hADSCs in vitro. Transplantation of NDNF‐overexpressing old hADSCs into the infarcted mouse hearts preserved cardiac function and reduced scar formation possibly through enhanced angiogenesis and arteriole genesis.

With the development of regenerative medicine technology, stem cell transplantation offers new treatment methods and new hope for heart diseases. Currently, stem cells have been investigated in the pre‐clinical and clinical studies, among which ADSCs are the most promising and potentially valuable source because of their large reserve, ease of access, high abundance, low immunogenicity, multi‐directional differentiation and strong self‐renewal ability. The International Federative Committee on Adipose Science points out that ADSCs are one of the ideal biological cells.^18^ ADSCs were first isolated and extracted from adipose tissue by Zuk et al^12^ These cells have the potential for multi‐directional differentiation, and the expression of surface markers conform to the criteria of stem cell identification.^19^, ^20^ In this study, adipose tissue was collected by abdominal open surgery from young and old patients. The surface markers identified by flow cytometry were consistent with the internationally recognized standard for the identification of ADSC,^21^, ^22^ and there were no statistical differences between the young and old group in the expression of these surface markers. These results suggest that age does not affect the expression of stem cell surface markers. Stem cells of different ages maintain good stem cell characteristics. However, the effect of age on the proliferation of ADSCs remains controversial. Previous research found that the proliferation of stem cells under the donor age of 20 was significantly higher than that from donors over 50 years old.^23^ However, Schipper et al 24 demonstrated that cell proliferation was significantly increased in the age range of 25–30 years old, but they were not significantly different in the age range of 40–45 years old or in the age range of 55–60 years old group. In vitro studies have shown that ADSCs have the potential of multi‐directional differentiation and are induced to differentiate into mature adipose cells, osteocytes, cardiac cells and vascular endothelial cells under certain conditions. Our experiment found that the abilities of adipogenic and osteogenic differentiation decreased significantly with age, which was consistent with the result from Ding et al,^25^ indicating that age is an important factor affecting the biological characteristics of stem cells. Although the separation and extraction of ADSCs are influenced by a number of factors such as donor sex, body mass index, source location, disease comorbidity, cell passage, cell cryopreservation and resuscitation, all of which may impact the experimental results,^26^, ^27^, ^28^, ^29^ age is still one of the key factors affecting the biological function of stem cells.

The mechanisms by which ADSCs treat MI are described to include possible cell differentiation into myocardial cells to repair the damaged heart tissue, but mainly act through paracrine effects. These include the promotion of cell proliferation and migration, facilitation of the neovascularization of the infarcted area, improvement in local blood supply, reduction of adverse ventricular remodelling, and maintenance of normal heart chamber size. Valina et al 30 found that after the transplantation of ADSCs into the infarcted area of pigs, a large number of endothelial and vascular smooth muscle cells could be seen in the infarct site, indicating that stem cell transplantation promotes angiogenesis and vascular genesis to provide better blood supply to myocardium and improves cardiac function. Fan et al 31 suggested that ADSCs enhance the signal of angiogenesis through mTOR and Akt, which are key regulatory factors in VEGF/mTOR/Akt pathway, thus ultimately achieving the effect of accelerating angiogenesis. Several clinical trials have suggested that treatment of acute MI and ischaemic cardiomyopathy with ADSCs improves left ventricular ejection fraction and reduces infarct area.^32^, ^33^ We speculate that treatment strategies with ADSCs that promote angiogenesis may provide new directions for cardiovascular diseases.

The neurotrophic factor NDNF is a secretory protein found in the nervous system, which is suggested to be involved in regulating nerve development, migration and differentiation, promoting hippocampal neuron migration and axon growth, and increasing neuronal survival.^15^, ^16^ Previous studies have shown that NDNF promotes endothelial angiogenesis and capillary regeneration, improves myocardial remodelling and function in mouse ischaemic hind limbs, strengthens the development of collateral circulation vessels, and has a beneficial effect on various ischaemic cardiovascular diseases.^34^, ^35^ This is consistent with the mechanism of action of ADSC transplantation for the treatment of MI. Previous research from our group has confirmed that the expression of NDNF in hMSCs was significantly reduced with age. Overexpression of NDNF in old hMSCs inhibited cell apoptosis, promoted angiogenesis and improved cardiac function.^17^ The present study found that NDNF expression in hADSCs was also negatively correlated with age. Furthermore, restoration of NDNF in old hADSCs improved biological function and rejuvenated the aged hADSCs. More abundant and better functional autologous hADSCs were obtained by amplification in vitro. After in vivo implantation of NDNF‐overexpressing old hADSCs, angiogenesis was facilitated which improved collateral circulation in the infarcted area and eventually led to the preservation in cardiac function and delaying the progression of heart failure.

## 5 CONCLUSION

The proliferation, migration and differentiation abilities of hADSCs decreased significantly with age. The level of NDNF in aged hADSCs was significantly lower than that in the young group, suggesting that NDNF may be used as a target for stem cell rejuvenation. Overexpression of NDNF in aged hADSCs significantly enhanced proliferation and migration in vitro as well as promoted local angiogenesis, repair and improved cardiac function after implantation into the infarcted mouse hearts in vivo. These findings provide experimental support for the clinical application of hADSCs in elderly patients with ischaemic heart disease.

## CONFLICT OF INTEREST

The authors confirm that there are no conflicts of interest.

## AUTHOR CONTRIBUTION

KY contributed to the collection of data, data analysis and interpretation, article writing; H‐FS, SH, W‐JY, X‐MF, FR, HG, X‐YZ, JZ, Z‐XP, and G‐XX contributed to the collection of data, data analysis and interpretation; JX contributed to financial support, conception and design and manuscript writing; R‐KL contributed to the administrative support, final approval of the manuscript.

## Data Availability

The data that support the findings of this study are available on request from the corresponding author.
