# Supplementary material for: Effect of neuron‐derived neurotrophic factor on rejuvenation of human adipose‐derived stem cells for cardiac repair after myocardial infarction
Source: J Cell Mol Med. 2019 Jul 9;23(9):5981–93. doi: 10.1111/jcmm.14456 (PMC6714174; doi:10.1111/jcmm.14456)
Supplement: Supplementary file 1 [file JCMM-23-5981-s001.docx]

**Supplemental Table 1. General characteristics of patients for *in vitro* and *in vivo* studies**

| **Variable** | **Age** | **N number** | **Male** | **Female** | **Disease** |
| --- | --- | --- | --- | --- | --- |
| ***In vivo*** |  |  |  |  |  |
| Young  (<40 years) | 30.86±4.45 | 7 | 2 (28%) | 5 (72%) | [Cholelithiasis](C:/Users/kun/AppData/Local/Youdao/Dict/Application/8.5.1.0/resultui/html/index.html#/javascript:;) (14%)  Abdominal trauma (14%)  [Caesarean](C:/Users/kun/AppData/Local/Youdao/Dict/Application/8.5.1.0/resultui/html/index.html#/javascript:;) [section](C:/Users/kun/AppData/Local/Youdao/Dict/Application/8.5.1.0/resultui/html/index.html#/javascript:;) (72%) |
| Old  (>60 years) | 72.14±9.65 | 7 | 3 (43%) | 4 (57%) | [Cholelithiasis](C:/Users/kun/AppData/Local/Youdao/Dict/Application/8.5.1.0/resultui/html/index.html#/javascript:;) (43%)Hydronephrosis (43%) [Intestinal](C:/Users/kun/AppData/Local/Youdao/Dict/Application/8.5.1.0/resultui/html/index.html#/javascript:;) [obstruction](C:/Users/kun/AppData/Local/Youdao/Dict/Application/8.5.1.0/resultui/html/index.html#/javascript:;) (14%) |
| ***In vitro*** |  |  |  |  |  |
| <40 years | 30.44±6.35 | 9 | 2 (22%) | 7 (78%) | [Cholelithiasis](C:/Users/kun/AppData/Local/Youdao/Dict/Application/8.5.1.0/resultui/html/index.html#/javascript:;) (11%)  Abdominal trauma (22%)  [Caesarean](C:/Users/kun/AppData/Local/Youdao/Dict/Application/8.5.1.0/resultui/html/index.html#/javascript:;) [section](C:/Users/kun/AppData/Local/Youdao/Dict/Application/8.5.1.0/resultui/html/index.html#/javascript:;) (67%) |
| 40-60 years | 51.4±4.84 | 10 | 4 (40%) | 6 (60%) | [Cholelithiasis](C:/Users/kun/AppData/Local/Youdao/Dict/Application/8.5.1.0/resultui/html/index.html#/javascript:;) (30%)  Abdominal trauma (20%)  Renal calculi (20%)  [Appendicitis](C:/Users/kun/AppData/Local/Youdao/Dict/Application/8.5.1.0/resultui/html/index.html#/javascript:;) (10%)  [Pancreatitis](C:/Users/kun/AppData/Local/Youdao/Dict/Application/8.5.1.0/resultui/html/index.html#/javascript:;) (10%)  Hydronephrosis (10%) |
| >60 years | 72.5±10.52 | 6 | 3 (50%) | 3 (50%) | [Cholelithiasis](C:/Users/kun/AppData/Local/Youdao/Dict/Application/8.5.1.0/resultui/html/index.html#/javascript:;) (50%) Hydronephrosis (33%)  [Intestinal](C:/Users/kun/AppData/Local/Youdao/Dict/Application/8.5.1.0/resultui/html/index.html#/javascript:;) [obstruction](C:/Users/kun/AppData/Local/Youdao/Dict/Application/8.5.1.0/resultui/html/index.html#/javascript:;) (17%) |
